# New acetogenin katsuurallene from *Laurencia saitoi* collected from Katsuura, Japan

**DOI:** 10.1007/s13659-022-00328-1

**Published:** 2022-03-10

**Authors:** Yu Minamida, Hiroshi Matsuura, Takahiro Ishii, Miyu Miyagi, Yuto Shinjo, Kosuke Sato, Takashi Kamada, Yoshihiro Mihara, Iwao Togashi, Keisuke Sugimoto, Tsuyoshi Abe, Norio Kikuchi, Minoru Suzuki

**Affiliations:** 1 Advanced Course of Applied Chemistry, National Institute of Technology, Asahikawa College, Shunkodai 2-2-1-6, Asahikawa, Hokkaido 071-8142 Japan; 2 Department of Materials Chemistry, National Institute of Technology, Asahikawa Collage, Shunkodai 2-2-1-6, Asahikawa, Hokkaido 071-8142 Japan; 3 Department of Biosciences and Biotechnology, Faculty of Agriculture, University of the Ryukyus, 1 Senbaru, Nishihara, Okinawa 903-0213 Japan; 4 Department of Materials and Life Science, Faculty of Science and Technology, Shizuoka Institute of Science and Technology, 2200-2 Toyosawa, Fukuroi, Shizuoka 437-8555 Japan; 5 Department of Medicinal Chemistry, Faculty of Pharmaceutical Sciences, Hokkaido University of Science, Maeda 7, 15-4-1, Teine-ku, Sapporo, Hokkaido 006-8590 Japan; 6 The Hokkaido University Museum, Hokkaido University, N10 W8, Kita-ku, Sapporo, Hokkaido 060-0810 Japan; 7 Coastal Branch of Natural History Museum and Institute, Chiba,, 123 Yoshio, Katsuura, Chiba 299-5242 Japan; 8 Present Address: Department of Life Science, Graduate School of Engineering Science, Akita University, 1-1 Tegatagakuen-machi, Akita, 010-8502 Japan

**Keywords:** *Laurencia*, Rhodomelaceae, Acetogenin, Triterpene, Diterpene, Biological activity

## Abstract

We examined the chemical constitution of the red alga *Laurencia saitoi* Perestenko, collected from Katsuura, Boso Peninsula, Chiba Prefecture, Japan. This specimen produced a new polyhalogenated acetogenin, named katsuurallene (**1**), whose structure was determined by the spectral methods, along with known diterpene, deoxyparguerol (**2**) and triterpene, thyrsiferol (**3**). In this paper we describe the structural elucidation of katsuurallene together with some biological activities.

**Supplementary Information:**

The online version contains supplementary material available at 10.1007/s13659-022-00328-1.

## Introduction

Red algae of the genus *Laurencia* (Rhodomelaceae, Ceramiales) are the most intensively studied of all algal genera. More than 1000 secondary metabolites with intriguing skeletal structures, including about 800 halogenated compounds, were reported from this unique genus [1–4]. New halogenated metabolites are being discovered from *Laurencia* [5, 6], which seem to be an endless source of novel compounds.

The *Laurencia* species, from which halogenated metabolites have been isolated, possess “*corps en cerise*” in both superficial cortical cells and trichoblast cells. “*Corps en cerise*”, an unusually swollen refractile inclusion, is recognized as the site of synthesis and/or storage of halogenated compounds [7]. On the other hand, some species without “*corps en cerise*” produce no halogenated compound. To date, the chemical constitution of 22 species of Japanese *Laurencia* with “*corps en cerise*”, including 7 taxonomically undescribed species, have been investigated [8–11].

As part of our additional studies of the chemical diversity in the Japanese *Laurencia* species, we examined the chemical composition of *Laurencia* spp. from Katsuura, Boso Peninsula, Chiba Prefecture. Boso Peninsula is situated in the southeast side of Kanto area on Honshu, the largest island of Japan. Its coastline faces Tokyo Bay to the west and Pacific Ocean to the east and south.

**Fig. 1:**
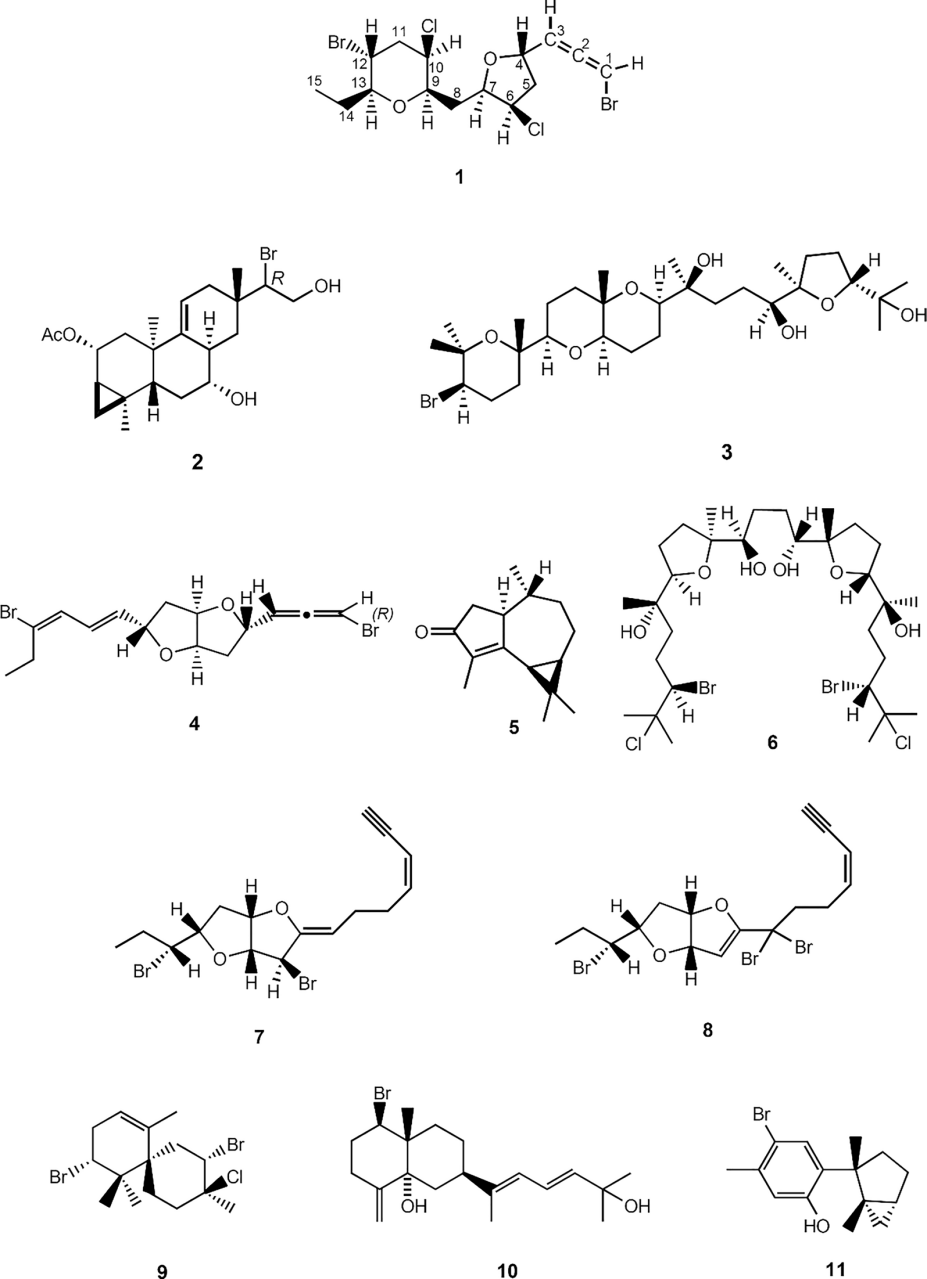
Chemical structures of **1**–**11**

The coasts of Katsuura are influenced by both the Kuroshio Current (warm current) and the Oyashio Current (cold current), and its marine flora contains more subtropical elements than subarctic elements. In the intertidal coast of Yoshio, Katsuura, four *Laurencia* spp., *L. saitoi*, *L. intricata*, *L. okamurae,* and *L. japonensis*, grow sympatrically from April and July [12]. Among them *L. intricata* contained zagashimallene (**4**), cyclocolorenone (**5**), and intricatetraol (**6**) [13]. *L. japonensis* contained two new brominated acetogenins katsuurenyne A (**7**) (Fig. 1) and katsuurenyne B (**8**) along with known 2,10-dibromo-3-chloro-α-chamigrene (**9**) and aplysiadiol (**10**) [14]. Furthermore, in the coast of Yoshio, *L. okamurae* unusually grow sympatrically in morphological variation, a clumpy type and a non-clumpy type. The extracts of both specimens showed almost identical patterns on TLC and contained laurinterol (**11**) as the major metabolite (unpublished result).

In this paper we describe the chemical composition of *Laurencia saitoi* and structure elucidation of a new polyhalogenated acetogenin, designated as katsuurallene (**1**), together with the biological activities of the isolated compounds.

## Results and discussion

### Chemical composition of *L. saitoi*

The EtOAc-soluble fraction was subjected to a combination of column and preparative thin-layer chromatography to yield a halogenated acetogenin, named katsuurallene (**1**), along with two known terpenoids, deoxyparguerol (**2**) and thyrsiferol (**3**).

Katsuurallene (**1**), [*α*]_D_^28^ + 73.5 (*c* 0.15; CHCl_3_), was analyzed for C_15_H_20_Br_2_Cl_2_O_2_ by HRESI-MS. The presence of a terminal bromoallenic side chain was proven by typical signals in the ^1^H- and ^13^C-NMR spectra (Table 1) [*δ*_H_ 6.09 (1H, *dd*, *J* = 6.0, 1.8 Hz) and 5.43 (1H, *dd*, *J* = 6.0, 6.0 Hz); *δ*_C_ 201.68 (C), 101.37 (CH), and 74.39 (CH)] [10]. Since the IR spectrum revealed no hydroxy and carbonyl absorptions, the two oxygen atoms in **1** were assumed to be involved in ether linkages.

**Table 1:** ^13^C (100 MHz; DEPT), ^1^H (400 MHz) NMR, and HMBC data for katsuurallene (**1**) (in CDCl_3_)

C^a^	^13^C (*δ*)	^1^H (*δ*)	Multiplicity, *J* (Hz)	Long-range correlations^d^ (H→C)
1	74.39	6.09	*dd*, *J* = 6.0, 1.8	C-2, C-3
2	201.68			
3	101.37	5.43	*dd*, *J* = 6.0, 6.0	C-2, C-4, C-5
4	73.79	4.89	*m*	C-2, C-7
5	42.96	~ 2.3	*m* (Ha)	
		~ 2.5	*m* (Hb)	
6	62.83	4.47	*dd, J* = 4.1, 4.1	C-4
7	77.94	4.26	*ddd*, *J* = 10.1, 2.8, 2.8	C-6, C-8
8	36.15	~ 1.7	*m* (Ha)	
		~ 1.9	*m* (Hb)	
9	76.05	3.88	*ddd, J* = 9.6, 2.3, 2.3	C-7, C-8, C-10, C-13
10	61.87	4.08^b^	*m*	C-11^e^, C-12^e^
11	43.96	~ 2.4	*m* (Ha)	
		~ 2.7	*m* (Hb)	
12	46.69	~ 4.10^c^	*m*	C-13^e^
13	83.79	3.41	*ddd, J* = 10.1, 9.2, 2.3	C-9, C-12, C-14, C-15
14	26.32	~ 1.5	*m* (Ha)	
		~ 2.0	*m* (Hb)	
15	9.72	0.98	*dd*, *J* = 7.3, 7.3	C-13, C-14

^a^Assigned by the HMQC spectrum

^b^*δ*_H_ 3.23 (*m*, *W*_*1/2*_ = 6.0 Hz) in benzene-*d*_6_

^c^*δ*_H_ 4.05 (*ddd*, *J* = 11.9, 10.1, 4.6 Hz) in benzene-*d*_6_

^d^Selected long-range correlations

^e^From the HMBC spectrum in benzene-*d*_6_

**Fig. 2:**
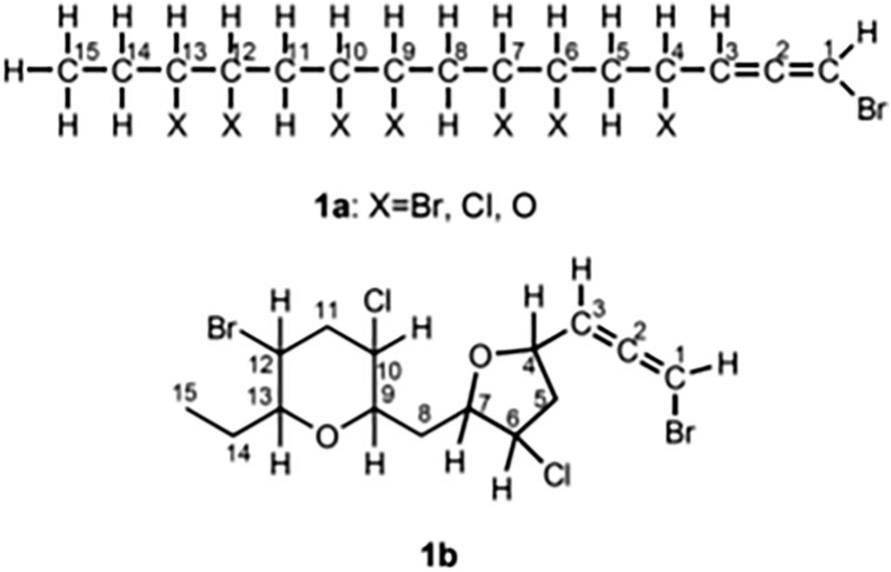
Partial structure **1a** and planar structure **1b**

Detailed analysis of the ^1^H- and ^13^C-NMR spectra, as well as HMQC and ^1^H-^1^H COSY spectra, led to the partial structure **1a** (Fig. 2) for katsuurallene (**1**). In **1a**, the oxygen atoms at C-4, C-7, C-9, and C-13 were verified based upon the chemical shift values of the pertinent carbons at 73.79 (C-4), 76.05 (C-9), 77.94 (C-7), and 83.79 (C-13), respectively. Moreover, the substituent at C-6 and C-10 were proven to be chlorine atom by the chemical shifts at 62.83 (C-6) and 61.87 (C-10). This was also confirmed by observation of the halogen-induced ^13^C isotope shifts [15] in the ^13^C-NMR spectrum. Therefore, the remaining bromine atom is attached to C-12.

The ^13^C-NMR spectrum showed that there were no other double bonds apart from those of the bromoallene moiety, and therefore katsuurallene (**1**), having four degrees of unsaturation, must be composed of two oxide rings. In the HMBC spectra of **1**, the long-range correlations between H-4/C-7, H-9/C-13 and H-13/C-9 (Table 1) were observed. This meant that two ether rings must be formed between C-4 and C-7 and between C-9 and C-13, leading to a planar structure **1b** for katsuurallene.

**Fig. 3:**
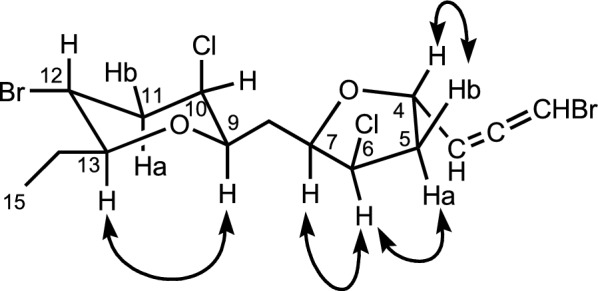
NOE correlations of **1**

The relative stereochemistry was partly determined as follows. In the NOESY spectrum of **1**, the nuclear Overhauser effect was observed between H-9 and H-13, thus indicating that both H-9 and H-13 have axial configurations on a tetrahydropyran ring with a chair-like conformation (Fig. 3). Furthermore, in the NMR spectrum (in C_6_D_6_) of **1** (Table 1, footnote), the H-12 showed the coupling constants of *J*_12,13_ = 10.9 Hz, *J*_11a,12_ = 11.9 Hz and *J*_11b,12_ = 4.6 Hz, which are typical axial/axial, axial/axial, and axial/equatorial coupling constants, respectively, indicating that the H-12 has axial configuration (equatorial bromine atom) on a tetrahydropyran ring. On the other hand, the H-9 showed the coupling constant of *J*_9,10_ = 2.3 Hz, which is a typical equatorial/axial coupling constant, indicating that the H-10 has an equatorial configuration (axial chlorine atom) on a tetrahydropyran ring.

The relative configuration on the oxolane ring was also determined by the NOESY spectrum. The *cis*-relationship of the substituents at C-6 and C-7 was shown by a NOE correlation between H-6 and H-7. H-6 was further correlated to Ha-5. On the other hand, Hb-5 was correlated to H-4, thus indicating the *trans*-relationship between H-4 and H-7 (Fig. 3).

In view of the positive sign of the optical rotation of **1**, the absolute configuration of the bromoallene moiety was suggested *S*-configuration, according to Lowe’s rule [16, 17], though a few exceptions to Lowe’s rule were reported in the case of microcladallenes [18] and also (*E*)- and (*Z*)-9-*epi*-omaezallene [10]. Consequently, the structure of katsuurallene would be represented by formula **1**.

**Fig. 4:**
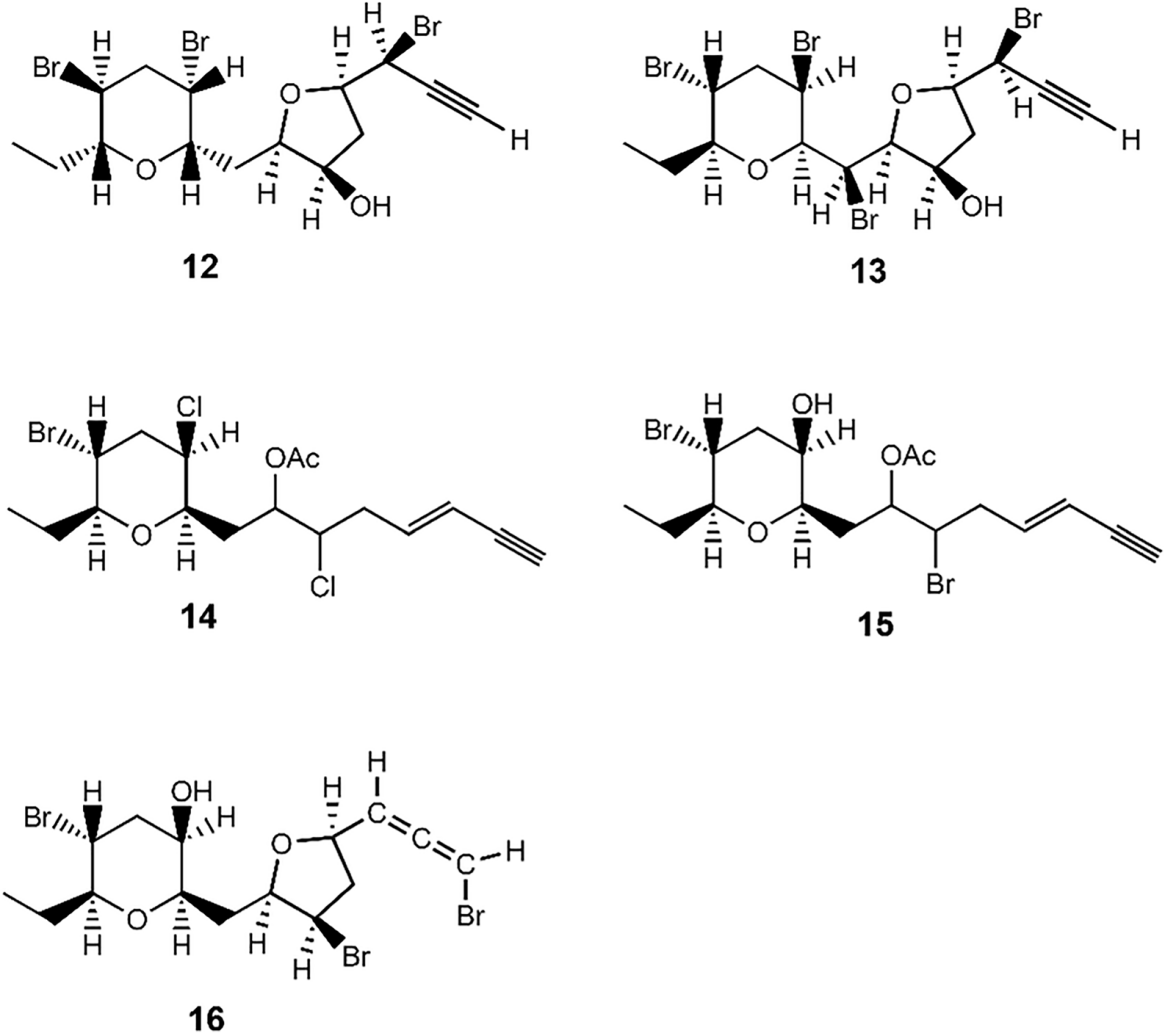
Chemical structures of **12**–**16**

As shown in Fig. 4, the related acetogenins have been found; **12** from *L. obtusa* (Canary Island) [19], **13** from *L. paniculata* (Turkey) [20] and bisezakyne-B (**14**), which may be a plausible shunt product of katsuurallene (**1**) biosynthetic pathway, from Japanese *Laurencia* sp. (Okinawa Prefecture) [15]. Furthermore, sargonenyne (**15**) and its related bromoallene (**16**) have been isolated from *L. obtusa* collected in Corsica [21, 22]. We are currently attempting to prepare a crystal suitable for X-ray crystallographic analysis in order to confirm the structure and establish the absolute stereochemistry for katsuurallene (**1**).

### Biological activity

Katsuurallene (**1**) was evaluated for insect repellent assay, *Arabidopsis* growth inhibition assay, antioxidant assay, brine shrimp assay and antimicrobial assay. Furthermore, zagashimallene (**4**), cyclocolorenone (**5**), and laurinterol (**11**) were evaluated brine shrimp assay, antioxidant assay and antimicrobial assay. **1** had weak toxicity for brine shrimp (LC_50_ = 855 μg/mL) and weak antimicrobial activity (no inhibition zone at 10 μg/disc, 11.0 mm inhibition ring at 30 μg/disc). **1** did not have insect repellent activity at 104 μg/cm^2^ (1.0 mg/disc), *Arabidopsis* growth inhibition activity at 100 μg/mL, and antioxidant activity at 100 μg/mL. Compounds **4**, **5** and **11** had toxicity for brine shrimp. LC_50_ of **4**, **5** and **11** were 3 μg/mL, 6 μg/mL and 37 μg/mL, respectively. Compounds **4**, **5** and **11** did not have antioxidant activity at 100 μg/mL and antimicrobial activity at 30 μg/disc.

### Conclusion

As described above, the specimen of *Laurencia saitoi* from Katsuura contained a new halogenated acetogenin, katsuurallene (**1**), as a characteristic major metabolite, along with deoxyparguerol (**2**) and thyrsiferol (**3**). On the other hand, the specimens from Teuri Island [23–27] and Suttsu [28] in Hokkaido contained diterpenes and triterpenes as characteristic metabolites. Since *L. saitoi* Perestenko has passed under the name *L*. *obtusa* (Hudson) Lamouroux in Japan [29], the former specimen from Teuri Island was first reported as *L. obtusa*.

Three Chinese specimens of *L. saitoi* have also been examined. The specimen collected from the coast of Yantai, Shandong Province, produced several parguerane-diterpenes and two triterpenes thyrsiferol and thyrsiferyl 23-acetate [30], which are very similar to the metabolites of the specimens from Hokkaido. However, the specimen collected from the coast of Rongcheng, northern Shandong Province, produced halogenated chamigrane-, bisabolane- and laurane-sesquiterpenes [31]. And the specimen collected from Hainan coastlines produced halogenated snyderane-sesquiterpenes [32]. The difference in chemical composition of the specimens of *L. saitoi* strongly requires doing taxonomical reexamination.

## Experimental

### General experimental procedures

IR spectra were recorded on a PerkinElmer FT-IR Spectrum Two spectrophotometer. ^1^H-NMR (400 MHz) and ^13^C-NMR (100 MHz) spectra were measured in CDCl_3_ or C_6_D_6_ by using JEOL-JNM-ECS-400 spectrometer. ESI-MS were obtained on a Hitachi High-Technologies Corporation NanoFrontier eLD spectrometer. Optical rotations were measured on a HORIBA SEPA-500 polarimeter. UV-Vis spectra were recorded on a JASCO V-650 spectrophotometer. Silica gel (Merck, Kieselgel 60, 70-230 mesh) were used for column chromatography (CC). Silica gel plate (Merck, Kieselgel 60 F_254S_) was used for preparative thin-layer chromatography (TLC).

### Plant material

*Laurencia saitoi* Prestenko was collected from the coast of Yoshio (35°8′N, 140°17′E), Katsuura, Boso Peninsula, Chiba Prefecture, on 18 May 2018. The voucher specimen has been deposited in the Herbarium of the Coastal Branch of Natural History Museum and Institute, Chiba (CMNH).

### Extraction and isolation of *L. saitoi*

The algal sample (58.0 g dry weight) was extracted twice with MeOH. The resulting MeOH solution was concentrated *in vacuo* and partitioned between EtOAc and H_2_O. The EtOAc layer was washed with water, dried over dry Na_2_SO_4_ and evaporated to leave an oily substance. The EtOAc-soluble extract (545 mg) was then fractionated by Si gel column chromatography with a step gradient (hexane and EtOAc). A portion (120 mg) of the fraction (158 mg) eluted with hexane–EtOAc (4:1) was further subjected to prep. TLC with toluene to yield a crude substance which was purified by prep. TLC with hexane-EtOAc (9:1) to give katsuurallene (**1**) (19.6 mg). A portion (85 mg) of the fraction (99 mg) eluted with hexane-EtOAc (1:1) was subjected to prep. TLC with hexane-EtOAc (1.5:1) to yield a crude substance which was further subjected to prep. TLC with toluene-EtOAc (2:1) to yield two fractions. The less polar fraction (25 mg) was then purified by prep. TLC with CHCl_3_-MeOH (95:5) to give deoxyparguerol (**2**) (6.3 mg). The polar fraction (30 mg) was purified by prep. TLC with CHCl_3_-MeOH (95:5) to give thyrsiferol (**3**) (7.7 mg).

Katsuurallene (**1**): Colorless solid; [*α*]_D_^28^ + 73.5 (*c* 0.15; CHCl_3_); IR ν_max_ (film) cm^−1^; 3057, 1960, 1372, 1309, 1196, 1090, 1026, 994, 819; ^1^H- and ^13^C-NMR spectra, Table 1 (Additional file 1: Figs. S1–S7); HR-ESIMS *m/z*; 460.9280. Calc. for C_15_H_20_^79^Br_2_^35^Cl_2_O_2_, 460.9285 [M + H]^+^.

Deoxyparguerol (**2**): Colorless oil; The ^1^H-NMR data (Additional file 1: Fig. S8) were found to be identical to those previously reported [33].

Thyrsiferol (**3**): Colorless solid; The ^1^H-NMR data (Additional file 1: Fig. S9) were found to be identical to those previously reported [34].

### Biological activity

#### Insect repellent assay

The repellent activities of some isolated compounds against the maize weevils *Sitophilus zeamais* were evaluated using the filter paper impregnation method as previously described [35]. The numbers of adult beetles present in each Petri dish were recorded after 24 h of exposure. Each treatment was repeated three times. Pyrethrin standard was used as a positive control.

#### Growth inhibition assay

The wild-type *Arabidopsis* seeds (Col-0) were immersed in 70% ethanol for 5 min and then 1.5% NaClO with Tween 80 for 5 min. Seeds were subsequently rinsed with distilled water, and then soaked in 0.1% agar solution for several hours at 4 °C. Surface sterilized seeds were grown in half-strength Murashige and Skoog (MS) medium supplemented with 0.8% agar and 1% sucrose for 1 week at 22 °C. For the plant growth assay, well-grown seedlings were selected and transferred to 12-well plate containing 1/2 MS medium with 1% sucrose. Test samples were dissolved in DMSO and prepared to a final concentration of 1 mg/mL (less than 1% DMSO). After the treatment of samples, the plates were incubated on a rotary shaker for 4 days at 22 °C under light–dark cycle conditions (12L:12D). The growth of the seedlings was estimated by the individual weights. All the experiments were performed three times.

#### Antioxidant assay

An aliquot of antioxidant Trolox or compounds diluted with ethanol (20 μL) was mixed with the 80 μM Tris–HCl buffer (pH 7.4) and then added to 100 μL of 200 μM 2,2-diphenyil-picrylhydrazyl (DPPH) (Alfa Aesar) in ethanol. The mixture was shaken vigorously and left to stand for 30 min at room temperature in the dark. The absorbance at 515 nm by DPPH was measured by UV–Vis spectrophotometer.

#### Brine shrimp assay

A bioassay of toxicity toward brine shrimp was performed as described in the literature [36]. Briefly, the compounds dissolved in ethanol were made up to 5, 10 and 50 μg/mL in artificial seawater. Serial dilution was made in the wells of 24-well microplates (Iwaki, Asahi Techno Glass Co., Tokyo, Japan) in triplicate in artificial seawater (2 mL). Brine shrimp eggs obtained locally (Japan Pet Design Co., Ltd., Tokyo, Japan) were hatched in artificial seawater at 25 °C. After 48 h, a suspension of nauplii containing 10–20 organisms (100 µL) was added to each well and incubated at 25 °C for 24 h and the numbers of non-motile and total nauplii in each well were counted in turn.

#### Antimicrobial assay

Antibacterial bioassays were carried out using *Escherichia coli* NBRC-3972 strain. Organism was precultured in LB medium for 2 days. The turbidity of the culture was adjusted to 10^7^ cells/mL using hemocytometer. Then 0.2 mL of the precultured bacterial suspension was used to seed LB agar plate. Paper discs (6 mm; ADVANTEC Toyo, Tokyo, Japan) impregnated with various amounts of the respective pure compounds were placed on the seeded agar plates and the diameters of the inhibitory zones were measured after the plates were incubated at 37 °C for 2 days.

## Supplementary Information

**Additional file 1. **^1^H-NMR (1D), ^13^C-NMR, DEPT, COSY, HMQC, HMBC, NOESY.
