# Correction: New acetogenin katsuurallene from *Laurencia saitoi* collected from Katsuura, Japan

**DOI:** 10.1007/s13659-025-00538-3

**Published:** 2025-08-29

**Authors:** Yu Minamida, Hiroshi Matsuura, Takahiro Ishii, Miyu Miyagi, Yuto Shinjo, Kosuke Sato, Takashi Kamada, Yoshihiro Mihara, Iwao Togashi, Keisuke Sugimoto, Tsuyoshi Abe, Norio Kikuchi, Minoru Suzuki

**Affiliations:** 1 Advanced Course of Applied Chemistry, National Institute of Technology, Asahikawa College, Shunkodai 2‑2‑1‑6, Asahikawa, Hokkaido 071‑8142 Japan; 2 Department of Materials Chemistry, National Institute of Technology, Asahikawa Collage, Shunkodai 2‑2‑1‑6, Asahikawa, Hokkaido 071‑8142 Japan; 3 Department of Biosciences and Biotechnology, Faculty of Agriculture, University of the Ryukyus, 1 Senbaru, Nishihara, Okinawa 903‑0213 Japan; 4 Department of Materials and Life Science, Faculty of Science and Technology, Shizuoka Institute of Science and Technology, 2200‑2 Toyosawa, Fukuroi, Shizuoka 437‑8555 Japan; 5 Department of Medicinal Chemistry, Faculty of Pharmaceutical Sciences, Hokkaido University of Science, Maeda 7, 15‑4‑1, Teine‑ku, Sapporo, Hokkaido 006‑8590 Japan; 6 The Hokkaido University Museum, Hokkaido University, N10 W8, Kita‑ku, Sapporo, Hokkaido 060‑0810 Japan; 7 Coastal Branch of Natural History Museum and Institute, Chiba, 123 Yoshio, Katsuura, Chiba 299‑5242 Japan; 8 Present Address: Department of Life Science, Graduate School of Engineering Science, Akita University, 1‑1 Tegatagakuen‑machi, Akita, 010‑8502 Japan

**Correction: Natural Products and Bioprospecting (2022) 12:10** 10.1007/s13659-022-00328-1

Following publication of the original article [1], the authors reported that the original version of this article unfortunately contained mistakes.

Page 1, section “Abstract”, the originally published texts were: This specimen produced a new polyhalogenated acetogenin, named katsuurallene (**1**), which structure was determined by the spectral methods, along with known diterpene, deoxyparguerol (**2**) and triterpene, thyrsiferol (**3**). The correct text should read: This specimen produced a new polyhalogenated acetogenin, named katsuurallene (**1**), whose structure was determined by the spectral methods, along with known diterpene, deoxyparguerol (**2**) and triterpene, thyrsiferol (**3**).

Page 2, the presentation of Fig. 1 was incorrect. The originally published Fig. 1 was:
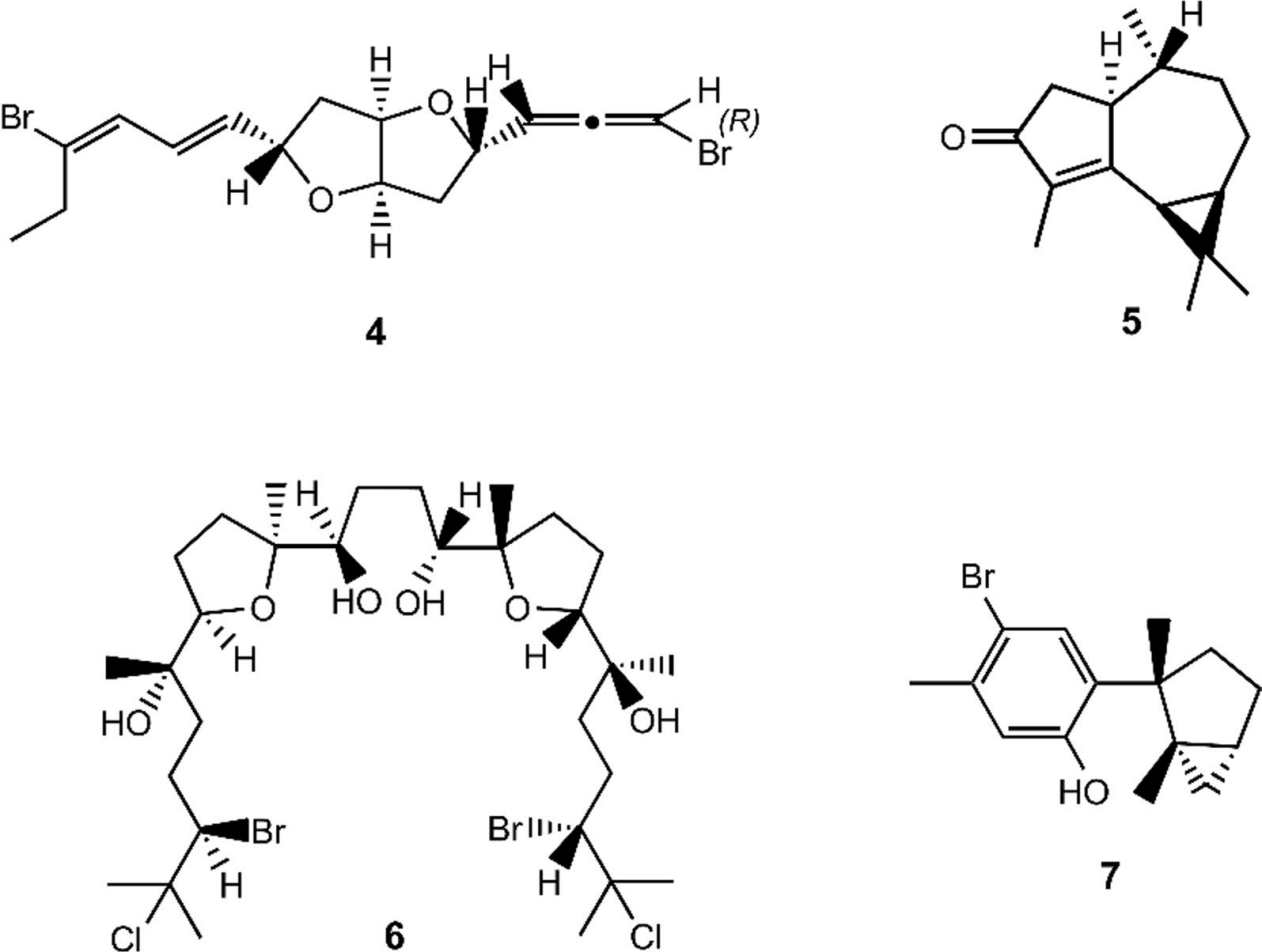


The corrected Fig. 1 is given below:

**Fig. 1:**
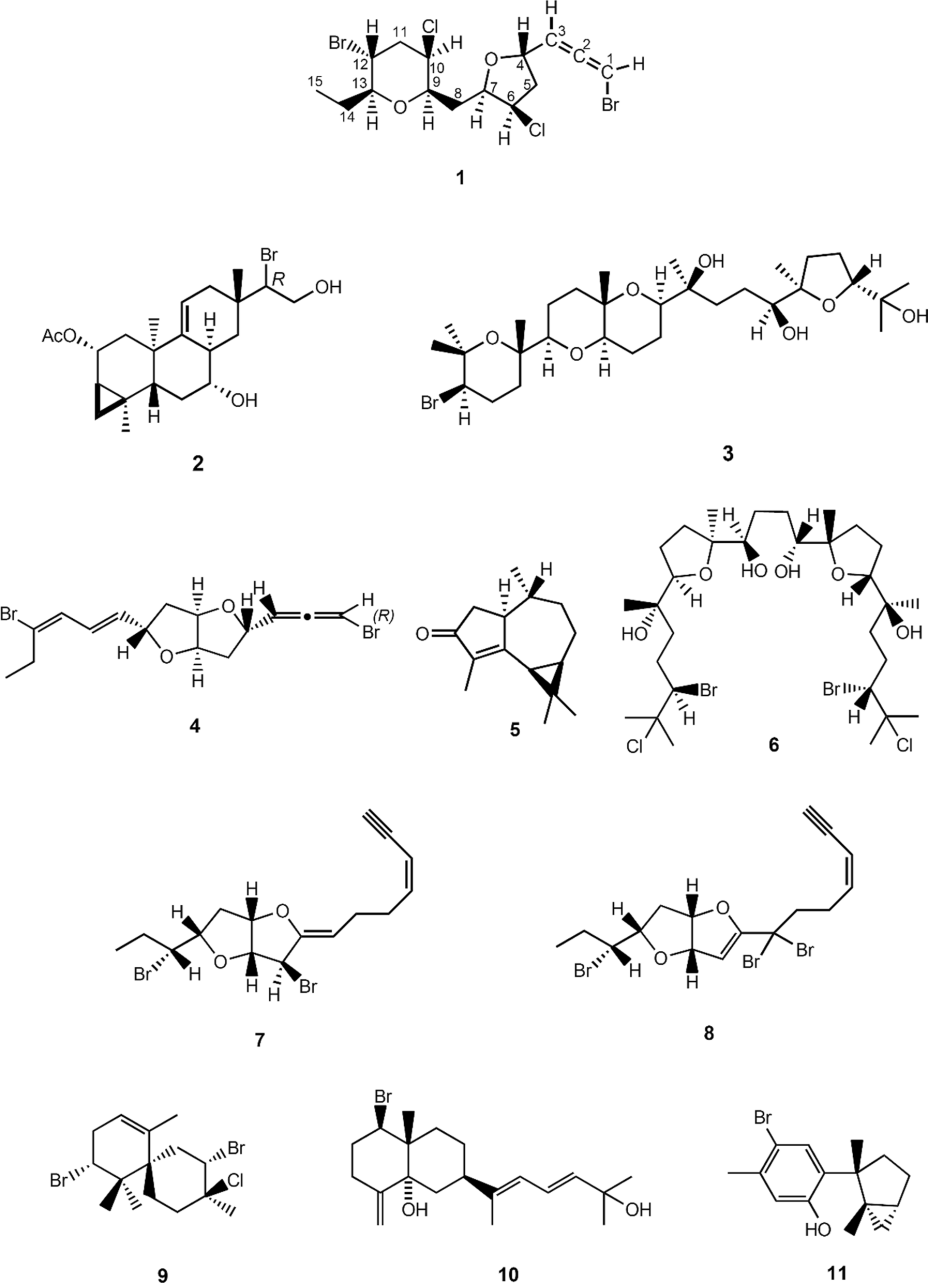
Chemical structures of **1–11**

Page 2, section “Results and discussion”, four paragraph, the originally published texts were: This meant that two ether rings must be formed between C-4 and C-7 and between C-9 and C-13, leading to a planar structure **1** for katsuurallene. The correct text should read: This meant that two ether rings must be formed between C-4 and C-7 and between C-9 and C-13, leading to a planar structure **1b** for katsuurallene.

Page 4, the presentation of Fig. 2 was incorrect. The originally published Fig. 2 was:
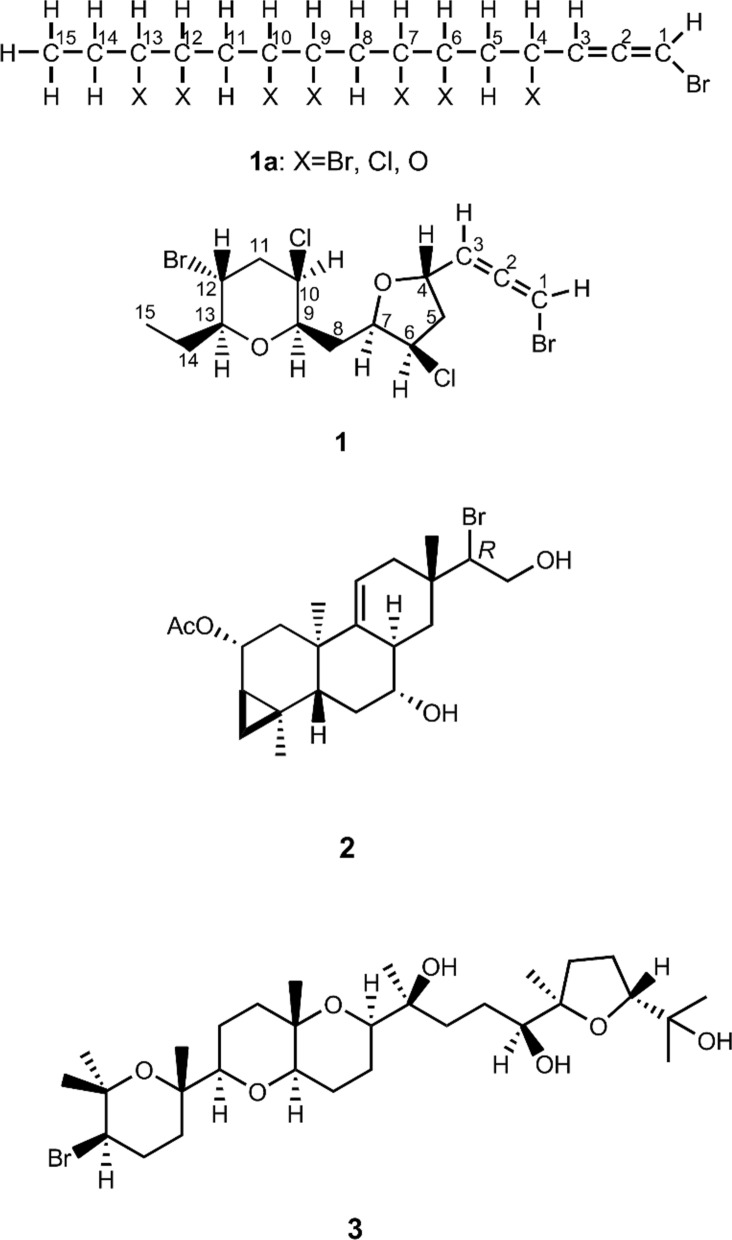


The corrected Fig. 2 is given below:

**Fig. 2:**
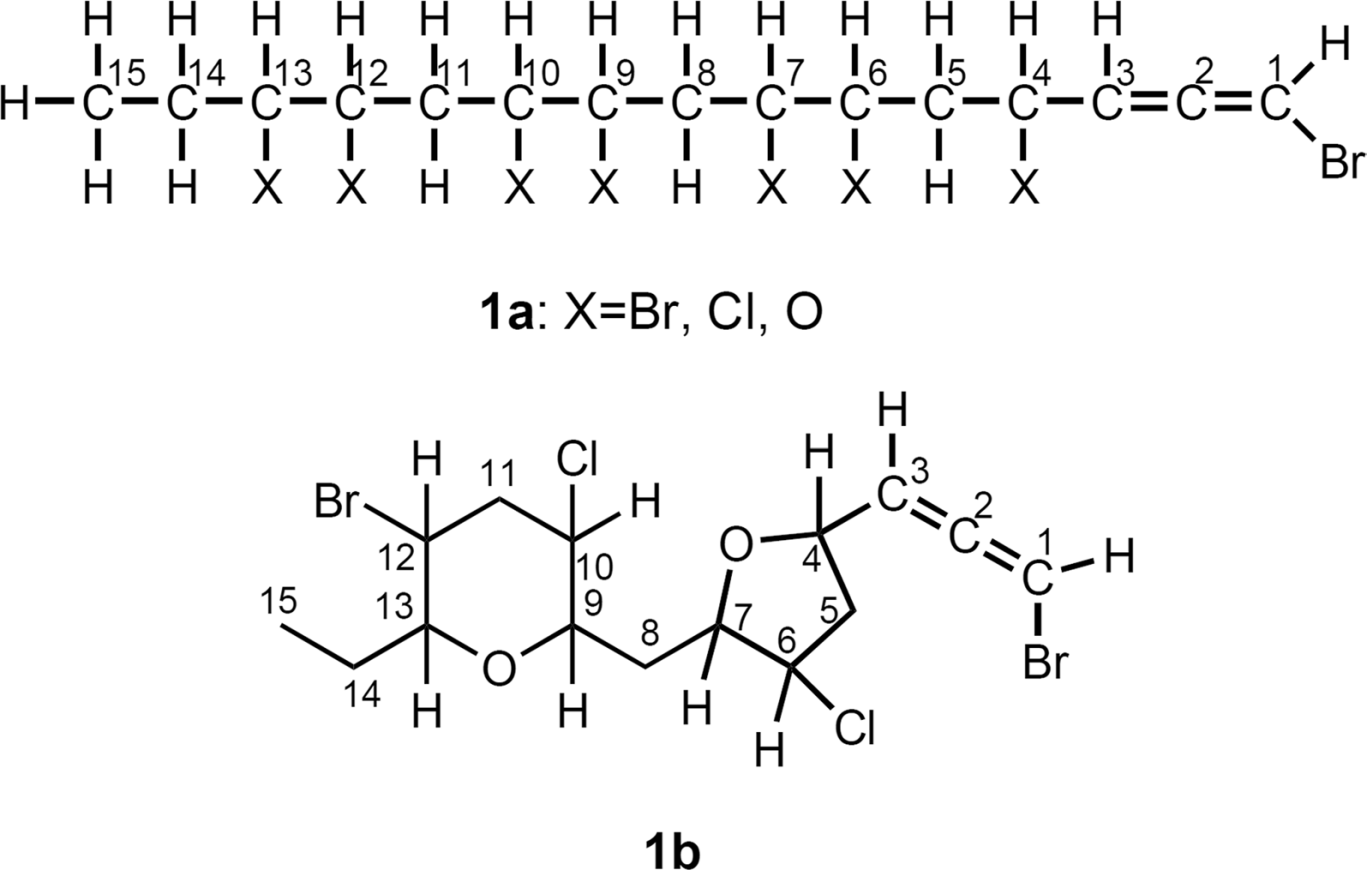
Partial structure **1a** and planar structure **1b**

Page 5, the presentation of Fig. 4 was incorrect. The originally published Fig. 4 was:
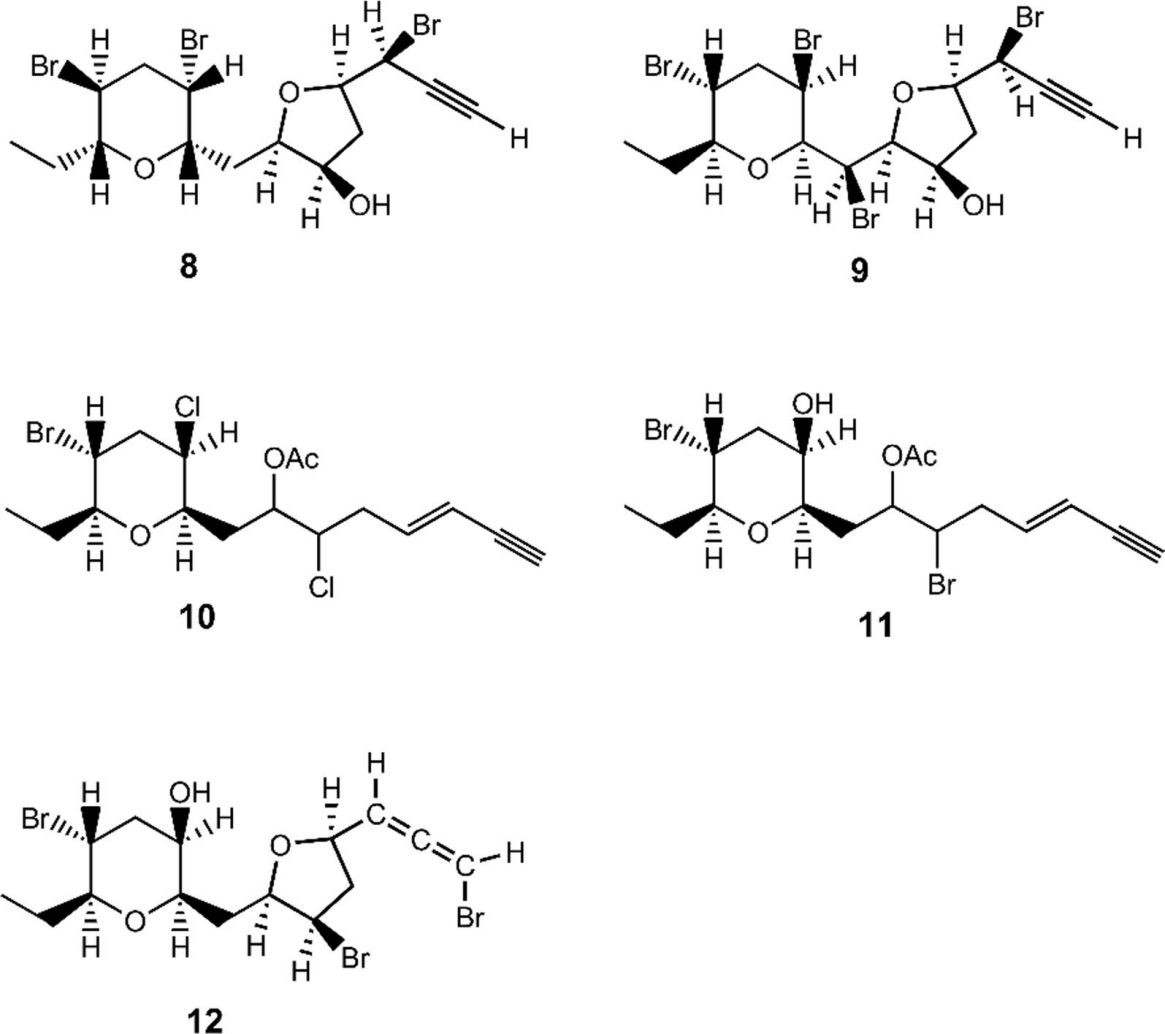


The corrected Fig. 4 is given below:

**Fig. 4:**
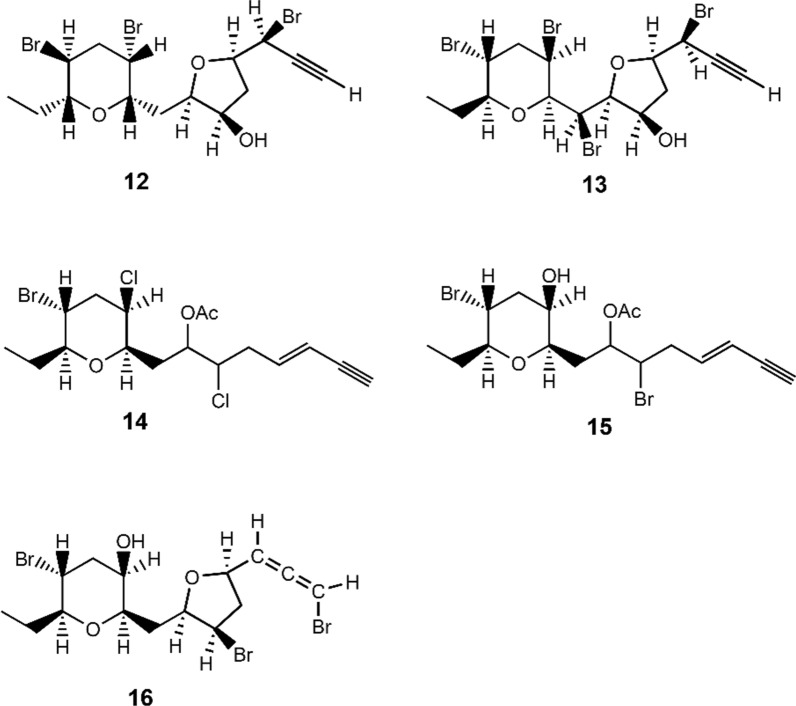
Chemical structures of **12–16**

The original article [1] has been updated.
