# Supplementary material for: New acetogenin katsuurallene from Laurencia saitoi collected from Katsuura, Japan
Source: Nat Prod Bioprospect. 2022 Mar 10;12(1):10. doi: 10.1007/s13659-022-00328-1 (PMC8907347; doi:10.1007/s13659-022-00328-1)

Supplementary material

New acetogenin katsuurallene from *Laurencia saitoi* collected from Katsuura, Japan

Yu Minamida, Hiroshi Matsuura*, Takahiro Ishii, Miyu Miyagi, Yuto Shinjo, Kosuke Sato, Takashi Kamada, Yoshihiro Mihara, Iwao Togashi, Keisuke Sugimoto, Tsuyoshi Abe, Norio Kikuchi, Minoru Suzuki

List of Figures

Figure S1. ^{1}**H NMR spectrum of Katsuurallene (1) in** $CDCl_3$ **(400 MHz).**

Figure S2. ^{13}**C NMR spectrum of Katsuurallene (1) in** $CDCl_3$ **(100 MHz).**

Figure S3. DEPT spectrum of Katsuurallene (1) in $CDCl_3$**.**

Figure S4. COSY spectrum of Katsuurallene (1) in $CDCl_3$**.**

Figure S5. HMQC spectrum of Katsuurallene (1) in $CDCl_3$**.**

Figure S6. HMBC spectrum of Katsuurallene (1) in $CDCl_3$**.**

Figure S7. NOESY spectrum of Katsuurallene (1) in $CDCl_3$**.**

Figure S8. ^{1}**H NMR spectrum of Deoxyparguerol (2) in** $CDCl_3$ **(400 MHz).**

Figure S9. ^{1}**H NMR spectrum of Thyrsiferol (3) in** $CDCl_3$ **(400 MHz).**

S1 ^{1}H NMR of Katsuurallene (**1**)

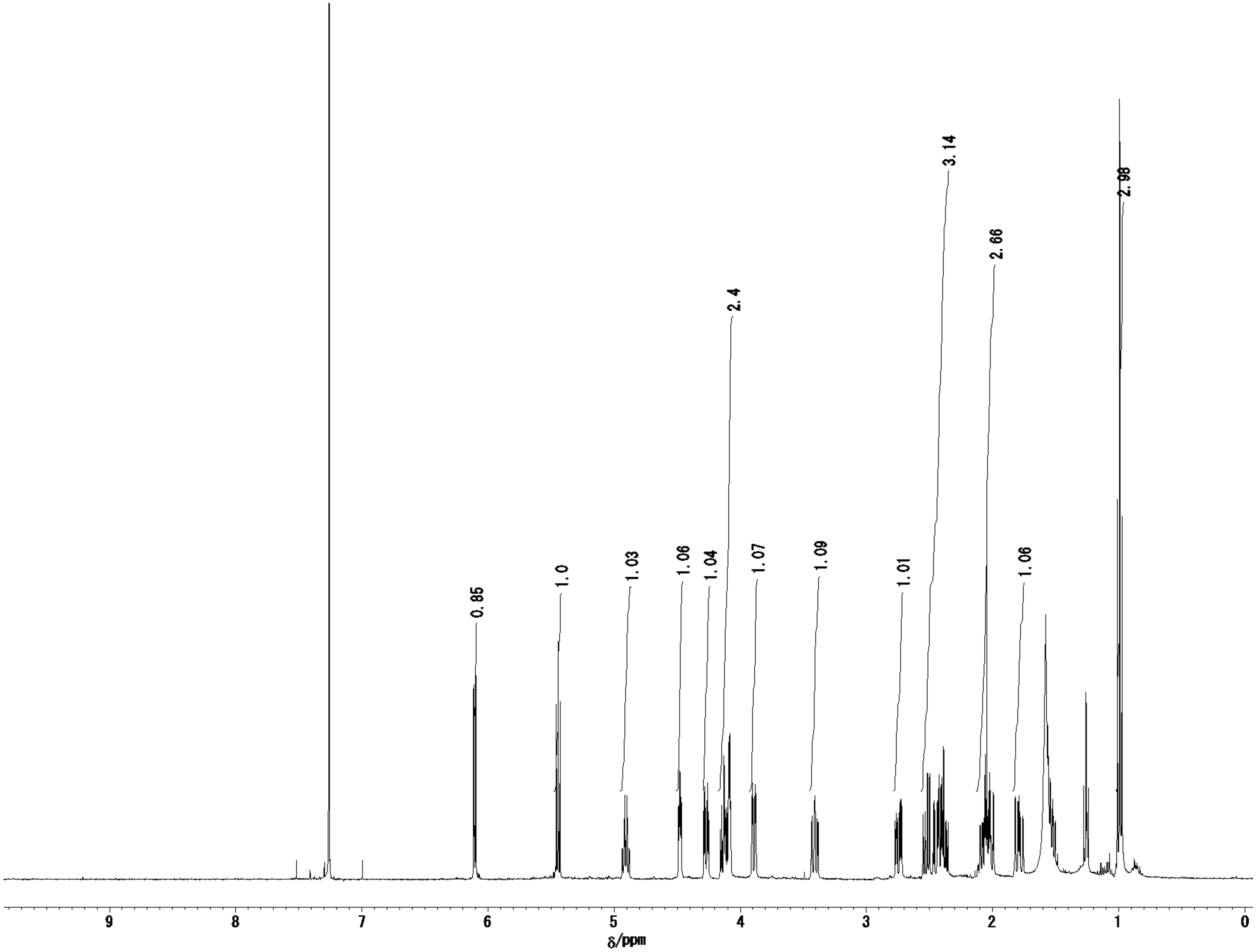

S2 ^{13}C NMR of Katsuurallene (**1**)

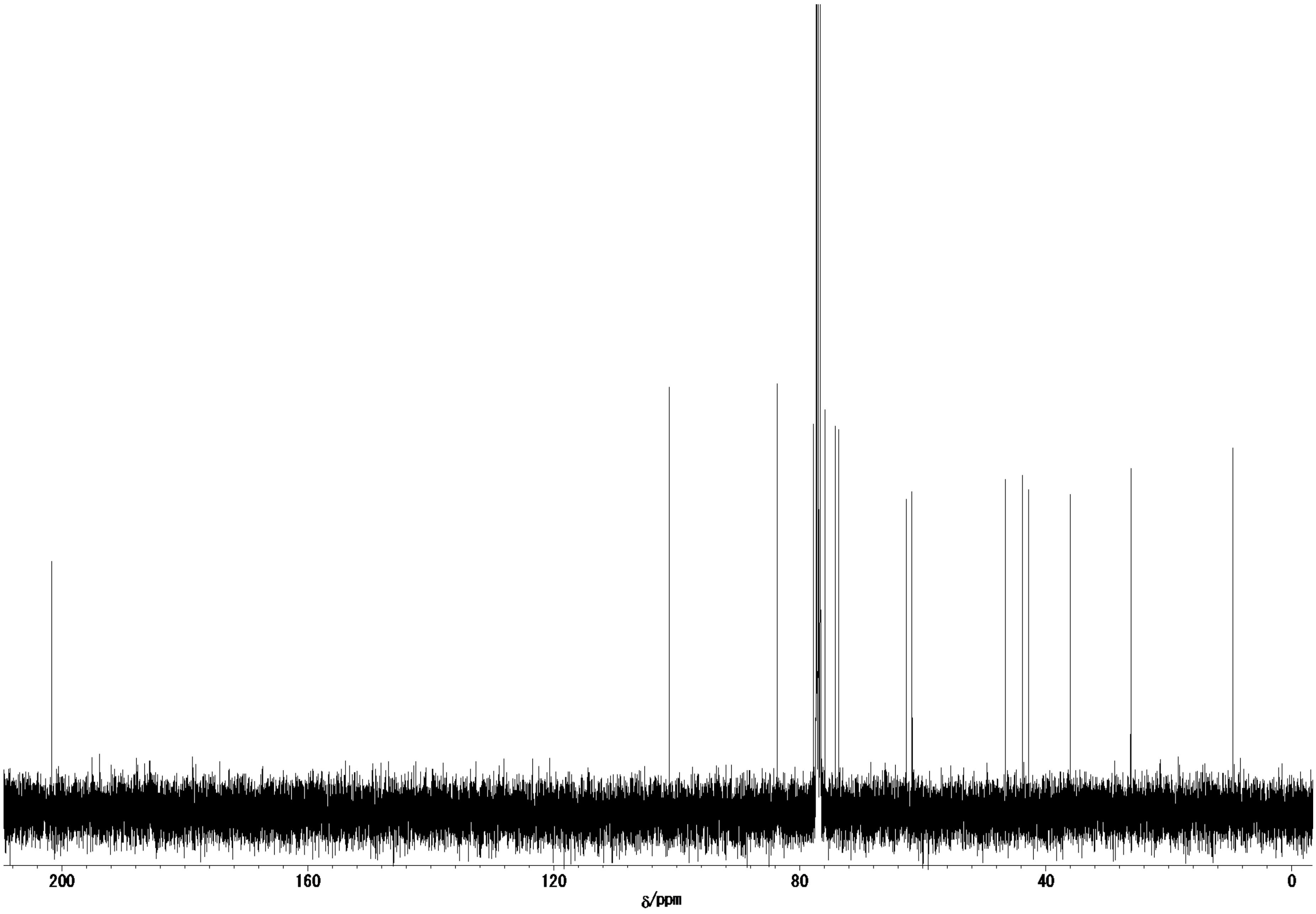

S3 DEPT of Katsuurallene (**1**)

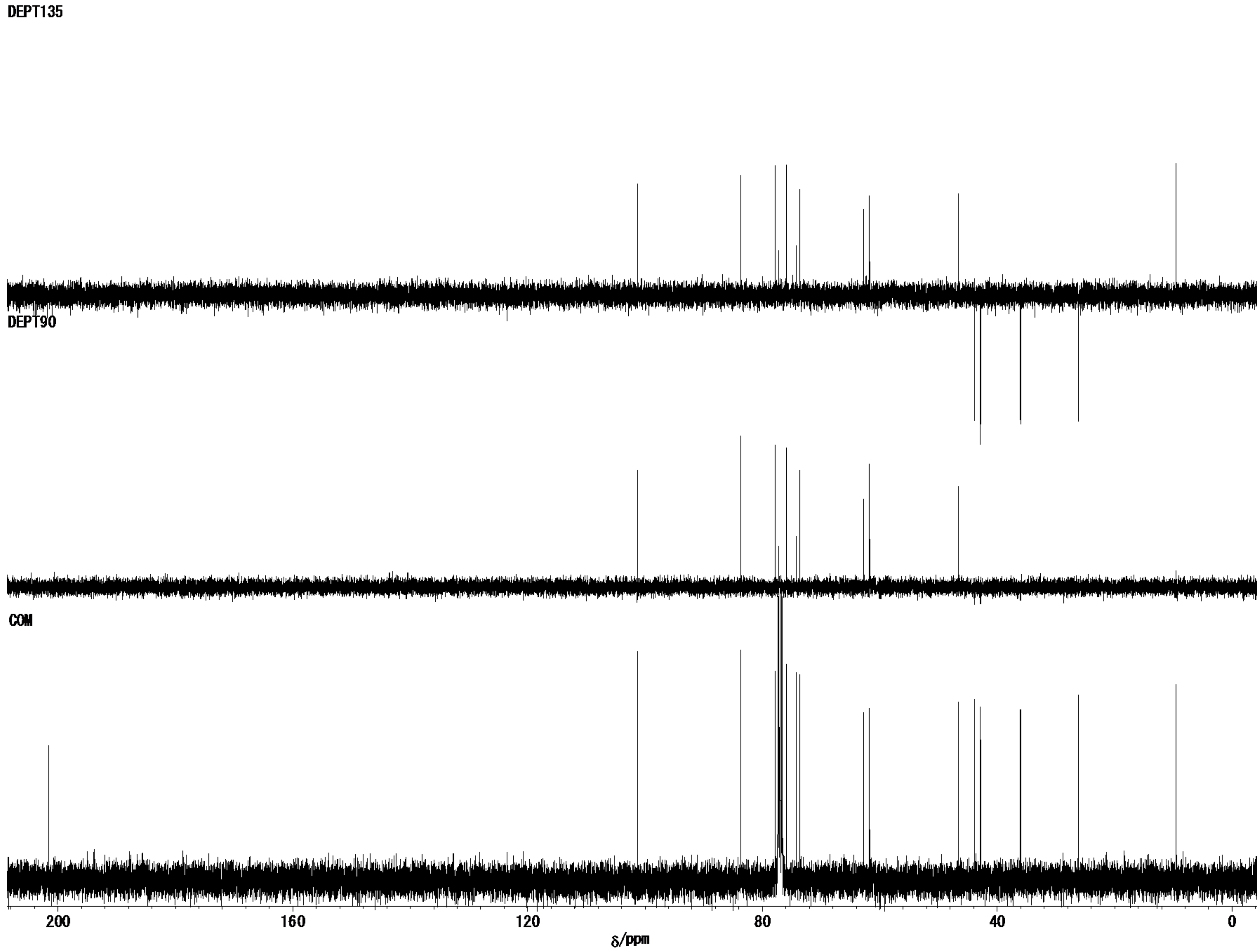

S4 COSY of Katsuurallene (**1**)

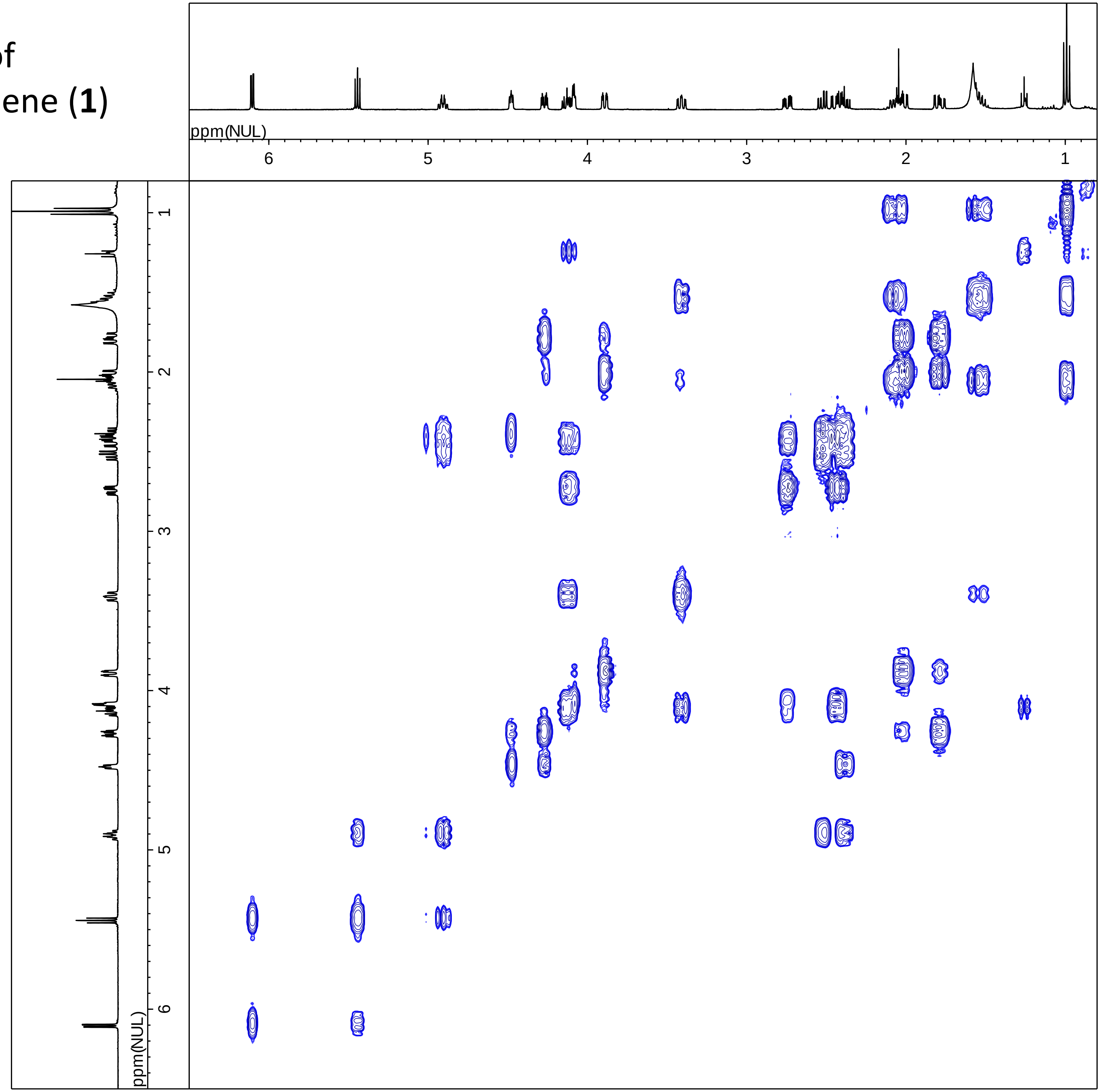

S5 HMQC of Katsuurallene (**1**)

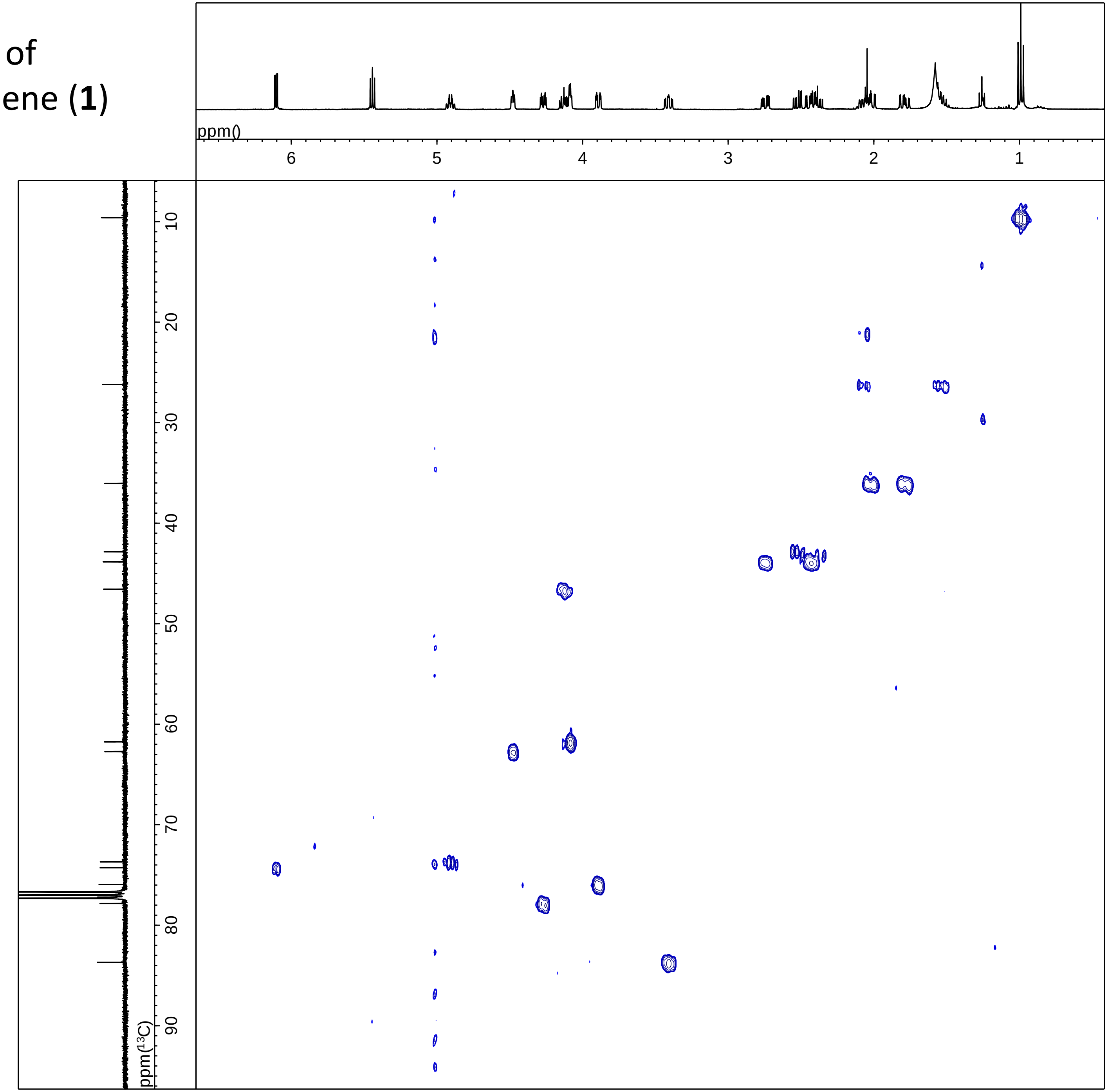

S6 HMBC of Katsuurallene (**1**)

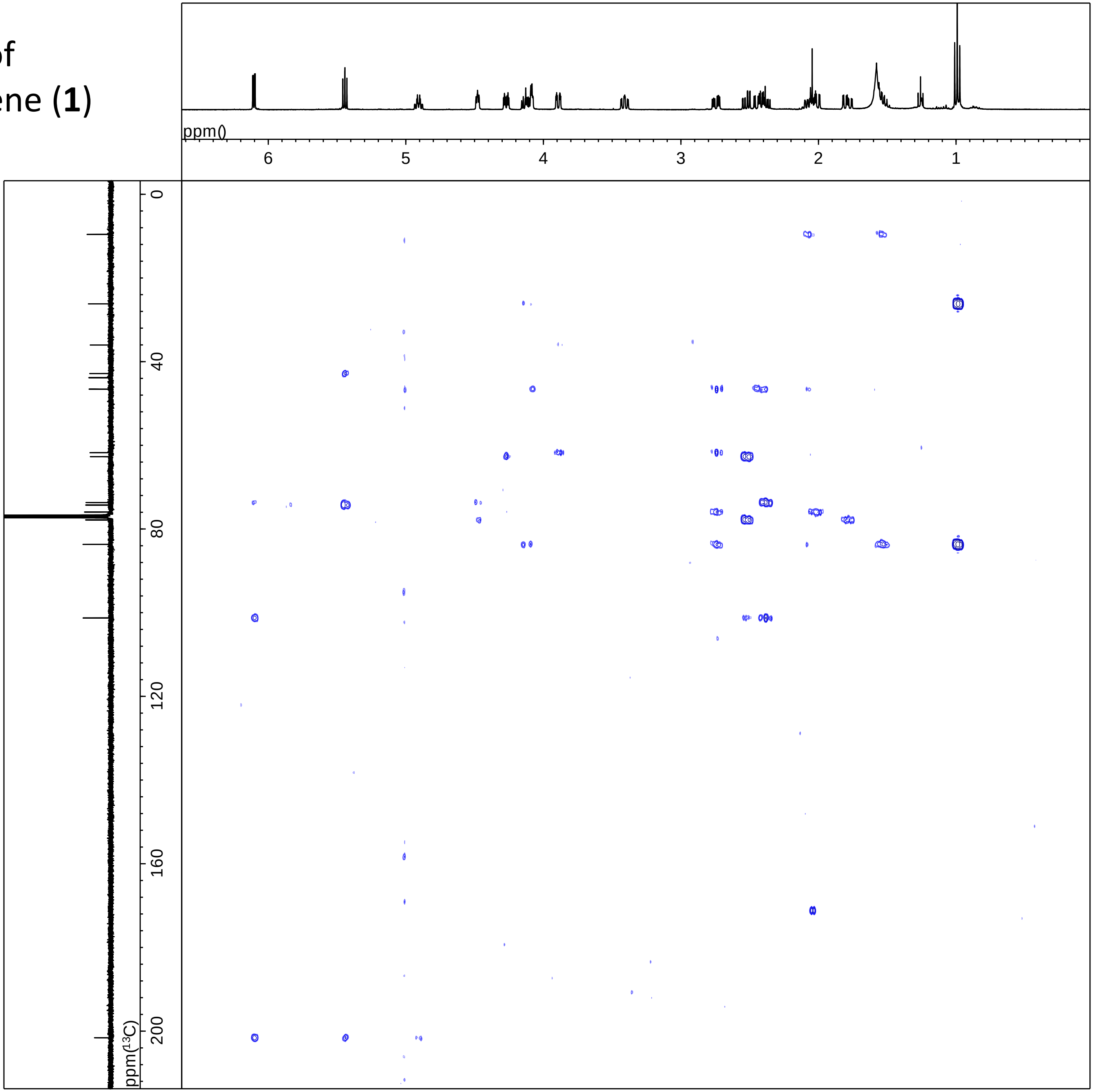

S7 NOESY of Katsuurallene (**1**)

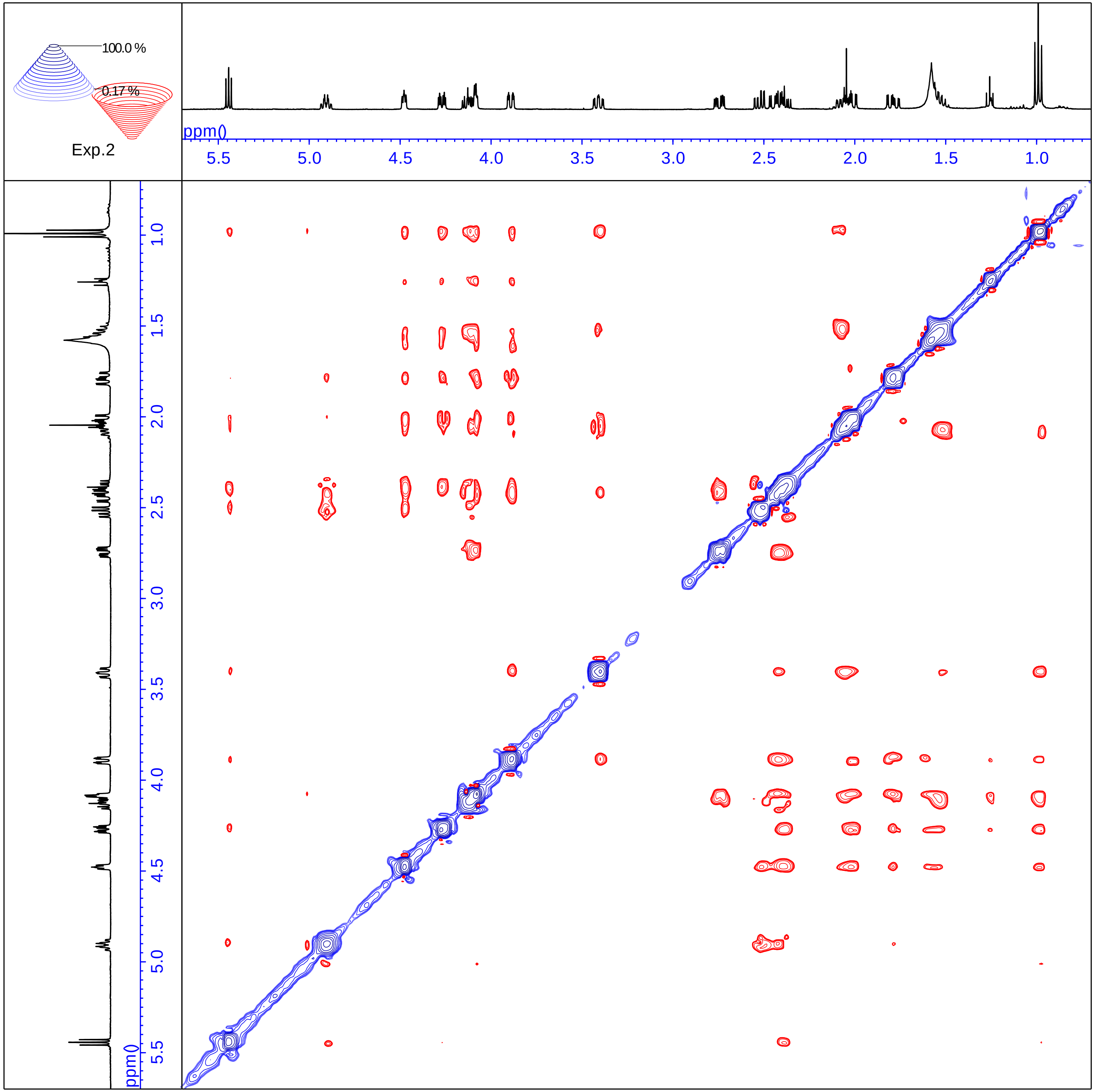

S8 ^{1}H NMR of Deoxyparguerol (**2**)

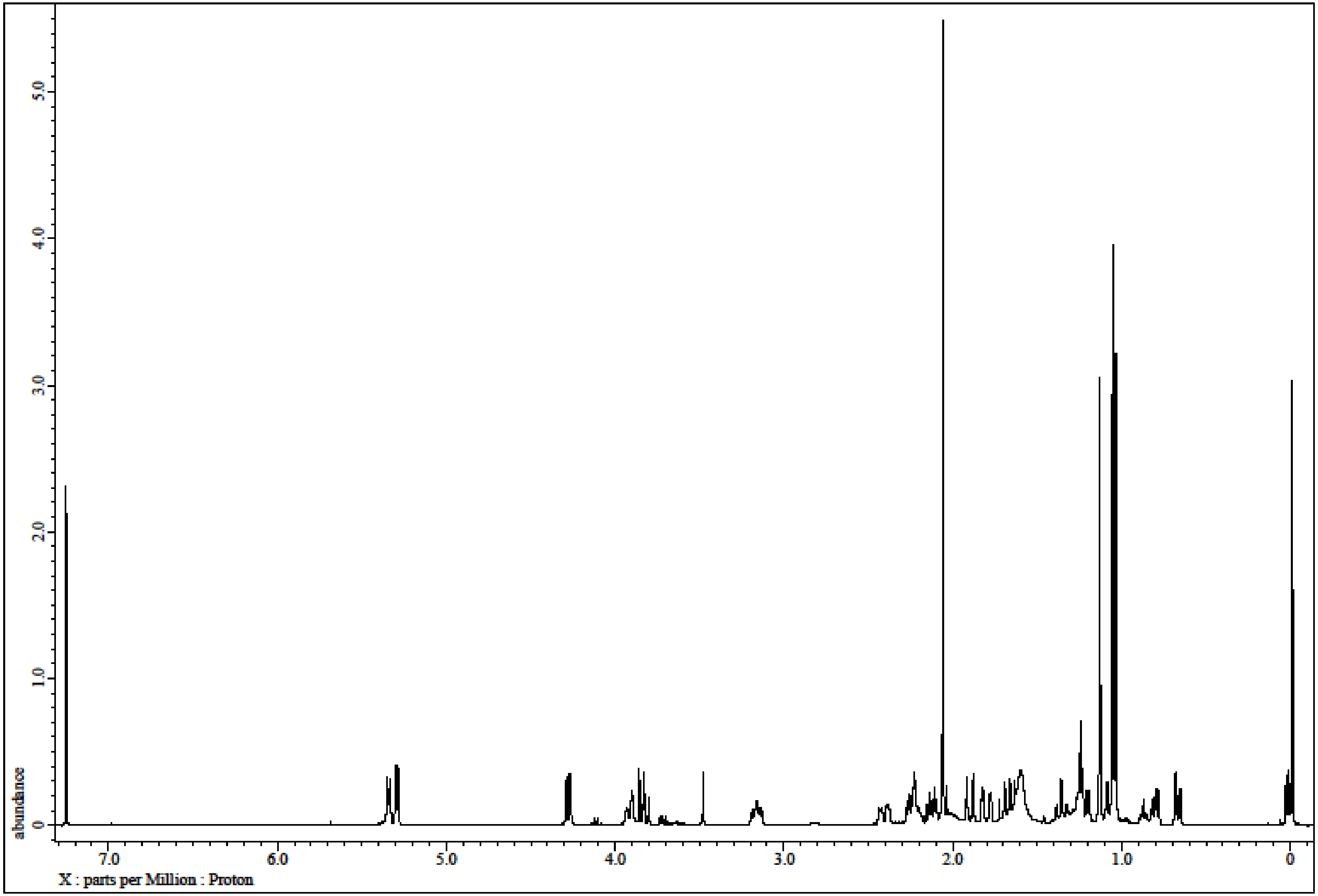

S9 ^{1}H NMR of Thyrsiferol (**3**)

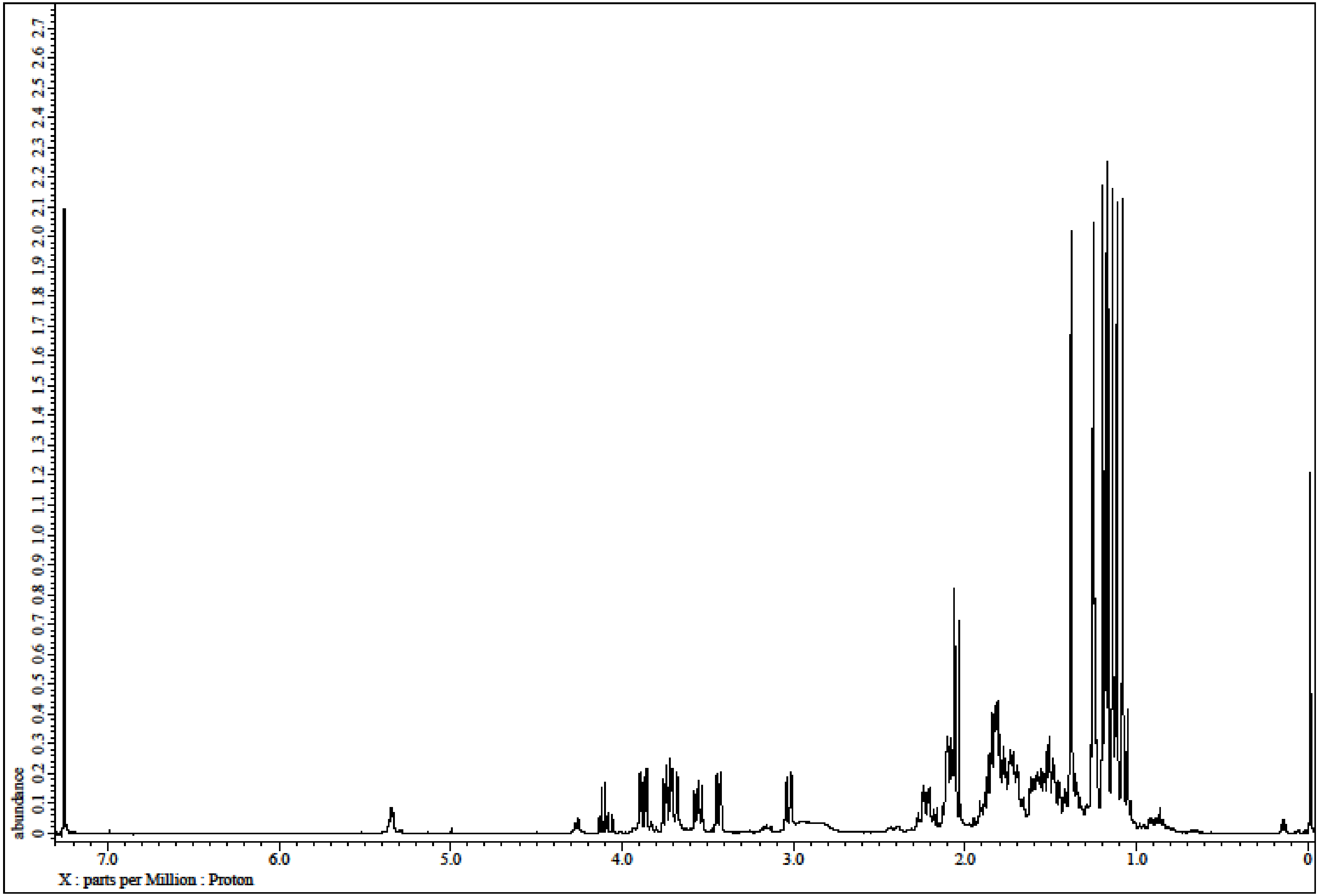

Supplement: Supplementary file 1 — Additional file 1. 1H-NMR (1D), 13C-NMR, DEPT, COSY, HMQC, HMBC, NOESY. [file 13659_2022_328_MOESM1_ESM.pdf]
